# Urgent Reversal of Direct Oral Anticoagulants in Critical and Life-Threatening Bleeding: A Multidisciplinary Expert Consensus

**DOI:** 10.3390/jcm13226842

**Published:** 2024-11-14

**Authors:** Anabela Rodrigues, Luciana Ricca Gonçalves, Tiago Gregório, Cilénia Baldaia, Gustavo C. Santo, João Gouveia

**Affiliations:** 1 Serviço de Imuno-Hemoterapia, Unidade Local de Saúde (ULS) Santa Maria, 1649-028 Lisboa, Portugal; 2 Serviço de Imuno-Hemoterapia, Unidade Local de Saúde (ULS) São João, 4200-319 Porto, Portugal; luciana.ricca@gmail.com; 3 Serviço de Medicina Interna e Unidade AVC, Unidade Local de Gaia e Espinho, 4434-502 Vila Nova de Gaia, Portugal; tiago.gregorio@ulsge.min-saude.pt; 4 CINTESIS—Centro de Investigação em Tecnologias e Serviços de Saúde, 4200-450 Porto, Portugal; 5 Serviço de Medicina Intensiva, Unidade Local de Saúde (ULS) Santa Maria, 1649-028 Lisboa, Portugal; cileniabaldaia@gmail.com (C.B.); joao.pereira.gouveia@ulssm.min-saude.pt (J.G.); 6 Serviço de Gastroenterologia, Unidade Local de Saúde (ULS) Santa Maria, 1649-028 Lisboa, Portugal; 7 Clínica Universitária de Gastrenterologia, Faculdade de Medicina de Lisboa, 1649-028 Lisboa, Portugal; 8 Serviço de Neurologia, Hospitais da Universidade de Coimbra, Unidade Local de Saúde (ULS) de Coimbra, 3004-561 Coimbra, Portugal; gustavo.cordeiro@ulscoimbra.min-saude.pt; 9 Center for Innovative Biomedicine and Biotechnology (CiBB), Universidade de Coimbra, 3004-561 Coimbra, Portugal; 10 Clínica Universitária de Medicina Intensiva, Faculdade de Medicina de Lisboa, 1649-028 Lisboa, Portugal

**Keywords:** direct oral anticoagulant, direct thrombin inhibitor, FXa inhibitors, life threatening, critical, bleeding, reversal, intracranial hemorrhage, trauma, gastrointestinal bleeding

## Abstract

Direct oral anticoagulants (DOACs) are increasingly being used due to their improved efficacy/safety ratio and lower clinical and economic burden when compared to vitamin K antagonists. However, bleeding is still the most frequent complication associated with DOACs, and although rare, bleeding episodes can be life-threatening or critical. The impact of DOAC anticoagulation activity during a bleeding event must be evaluated according to patient clinical assessment, dosage and time from last intake, the presence of comorbidities (especially kidney and liver dysfunction), and, whenever possible, coagulation tests. Unfortunately, DOACs’ anticoagulation activity is not easily or usually detectable in routine common coagulation testing. Specific DOAC tests allow for specific drug monitoring, but they are too time consuming, and are usually unavailable in routine emergency practice. If a clinically relevant DOAC plasma concentration is assumed or proven in a severe bleeding scenario, DOAC reversal is needed to restore hemostasis. This experts’ consensus provides a narrative review about DOAC reversal and practical life-threatening bleeding management in several scenarios (trauma, intracranial hemorrhage and gastrointestinal bleeding), focusing on the selection of patients to whom specific reversal agents should be given.

## 1. Introduction

Anticoagulation therapy has increasingly been used for the treatment of several cardiovascular conditions, such as the prevention of stroke and systemic embolism in atrial fibrillation (AF) patients and patients with a mechanical heart valve, as well as the prevention and treatment of venous thromboembolism (VTE) and acute coronary syndromes [1,2,3]. It has also been used in patients with liver cirrhosis and AF [4]. The use of direct oral anticoagulants (DOACs) has increased significantly in recent years (doubling between 2017 and 2020 [5]) with decreasing use of vitamin K antagonists (VKAs) since 2011 [6,7]. About 2% of the United States’ and Occidental Europe’s populations use a factor X-activated (FXa) inhibitor [8], and this use is expected to increase according to several scientific societies [9,10,11].

Currently, the guidelines recommend the use of DOACs over VKAs for the prevention of stroke and systemic embolism in patients with non-valvular AF, for the prevention and treatment of VTE [2,6,12], and for the secondary prevention of arterial ischemic events in patients with chronic coronary or peripheral artery disease [12], due to their safety and efficacy profile [13,14,15].

DOACs act as anticoagulants by inhibiting specific serine proteases and include FXa inhibitors (apixaban, edoxaban, rivaroxaban, and betrixaban) and thrombin inhibitors (dabigatran). They present an improved efficacy/safety ratio with a significant reduction in critical bleeding, namely intracranial hemorrhage (ICH), and have a predictable anticoagulant effect without the need for routine monitoring and fewer food and drugs interactions when compared with VKA, and they can be administered in fixed-dose regimens according to indications, patient characteristics (age, body weight, renal function), and the use of concomitant drugs [1,2,4,5,6,14,16,17,18].

As anticoagulation becomes more common, associated bleeding events are expected to increase [1,5], especially due to increasing age, comorbidities, overall fragility, and increasing AF prevalence [13,16]. Hemorrhage is by far the most frequent complication of anticoagulant therapy [19], and remains a major problem [1]. While DOACs have lower bleeding rates compared with VKA, the risk of bleeding complications remains, and there is a critical need for DOAC reversal in patients with life-threatening or severe bleeding, or those requiring urgent surgeries or procedures associated with a high risk of bleeding in an emergency setting, to avoid exsanguination and to reduce mortality [1,10,17,20,21]. The annual rates of major bleeding and ICH are 2–3.5% and 0.3–0.5%, respectively [8,22], in patients with AF receiving DOAC. The in-hospital mortality rate is about 26–30% in patients receiving FXa inhibitors presenting with spontaneous ICH [22,23].

The top three most common types of bleeds leading to hospitalization are ICH, trauma-related bleeding, and gastrointestinal bleeding (GIB). When these three scenarios occur in patients on DOAC therapy, effective and fast support from the hospital team is urgently needed. This need serves as the foundation for developing an algorithm for urgent DOAC reversal in cases of critical and life-threatening bleeding.

## 2. Material and Methods

Six physicians with different areas of expertise (two transfusion medicine/hemostasis specialists, one neurologist, one gastroenterologist, one intensive care medicine specialist, and one internal medicine specialist) from different hospitals gathered to develop an algorithm for DOAC reversal in the context of life-threatening and/or critical bleeding.

The most recent guidelines concerning DOAC reversal in the context of bleeding were reviewed. The following search terms were used on the PubMed Google Scholar, Medline, and Research Gate databases: direct oral anticoagulant, direct thrombin inhibitor, FXa inhibitors, anticoagulation guidelines, atrial fibrillation, life-threatening, critical bleeding, bleeding, DOAC reversal, intracranial hemorrhage, trauma, gastrointestinal bleeding. The results were screened by title and abstract analysis to assess their relevance. We mostly selected articles published since 2014, including guidelines (national and international), experts’ statements, clinical studies (pivotal and real-world studies), and case reports about specific and rare clinical situations. From a total of 140 manuscripts, the most recent 125 were chosen for inclusion in this review, representing the most recent research in the field in the last 10 years.

## 3. Relevant Sections

### 3.1. Definitions of Major, Life-Threatening, and Critical Bleeding

Bleeding evaluations should address life-threatening situations and critical site events, as well as the rate and volume of blood lost [5,24]. The definitions of life-threatening bleeding events or critical sites may vary significantly, particularly when these terms are used in clinical trials [5,13,24,25,26]. Massive bleeding is usually defined according to the volume of blood lost, the ongoing hemorrhage rhythm, and the number of transfused units of red blood cells (RBC), as specified in Table 1 [27,28,29,30,31].

In clinical practice we may use the pragmatic definition of bleeding associated with a shock index (heart rate/systolic blood pressure ratio) ≥1.0 to identify a massive hemorrhage scenario [25,27,28,29]. Hemoglobin (Hb) also can be used to predict the significance of blood loss [24]. However, the time lag between the bleeding and Hb fall due to hemoconcentration may overlook the magnitude of bleeding [24].

Critical bleeding is defined as major life-threatening bleeding or a hemorrhage with a smaller volume in a critical area (e.g., intracranial) or organ, resulting in morbidity and death [29]. Critical sites are based on space-occupying lesions and the predicted morbidity and mortality of hemorrhage (e.g., brain, spine) [5].

The International Society of Thrombosis and Hemostasis (ISTH) and the Subcommittee on Control (SCC) of Anticoagulation recommend the criteria defined in Table 2 for defining major bleeding in non-surgical patients [19,32].

The ISTH also defined a prognostic classification for major bleeding to assess the risk of short-term death within 30 days while on oral anticoagulants, as presented in Table 3 [33]. According to the ISTH analysis, a fall in Hb levels of ≥2 g/dL or the transfusion of 2 units of RBC was not a predictor of death [33].

### 3.2. Evaluation of Conditions to DOAC Reversal

#### 3.2.1. Patient’s Clinical Condition

Patient’s age, weight, organ dysfunctions (e.g., kidney/liver failure), frailty, indication for anticoagulation, and previous thrombotic or bleeding events will directly affect the clinical course and can impact the efficacy and safety of DOAC reversal therapy [24,34]. Pharmacological findings are also directly related to the time elapsed after ingestion [24]. As a general principle, when five half-lives have passed from the last dose of DOAC, the effects are considered to be completely reversed [24]. However, if the estimated glomerular filtration rate (eGFR) is under 30 or 50 mL/min, the DOAC effect may still exist.

Most bleeding complications associated with DOACs can be managed by withholding the anticoagulant and providing supportive measures to preserve the patient’s hemodynamic stability and enhance their renal excretion of the drug. However, if urgent anticoagulation reversal is required, other measures must be taken and should be chosen according to the clinical situation. Recently, from a retrospective study of 245 trauma patients treated with apixaban and rivaroxaban with anti-Xa levels measured at admission, a significant variability in their therapeutic anti-Xa activity thresholds was confirmed [35]. Female sex, increased age, decreased height and weight, and lower estimates of creatinine clearance (or eGFR) were associated with higher anti-Xa levels at admission [35]. The authors of this study were also unable to detect an association between anti-Xa levels and clinical outcomes; specifically, there was no correlation between anti-Xa level and the need for transfusion or the administration of a reversal agent, a diagnosis of ICH on admission, progression of ICH, or length of hospital stay or mortality [35].

#### 3.2.2. DOAC Pharmacokinetics

DOACs present favorable pharmacokinetic characteristics, with a rapid onset (within 2–4 h) and relatively short half-lives (half-lives between 5 and 14 h for FXa inhibitors and 14 and 17 h for dabigatran in patients with normal kidney function) [17,22]. Renal clearance is higher in patients treated with dabigatran (80%) than in FXa inhibitors (edoxaban, 50%; ribaroxaban, 36%; apixaban, 27%) [17,36].

A high plasma concentration can be assumed if DOAC intake occurred within the last 6–8 h, and relevant concentrations can be assumed if the intake was in the last 12–18 h [36,37,38]; most anticoagulant activity is gone in 24 h, unless eGFR is below 30 mL/min, which is particularly common with dabigatran treatment [17,36,37,39]. However, it may last for up to 48 h or more in those with impaired renal function [22]. The risk increases with age, overdosing, and the degree of renal impairment [36].

#### 3.2.3. DOAC Assays

Routine coagulation monitoring is usually not required for DOACs [40]. However, an assessment of their anticoagulant effect may be necessary in some clinical settings, such as in patients presenting with acute major bleeding or thrombotic events, or prior to urgent invasive procedures. DOACs interfere with most clot-based hemostasis tests, like prothrombin time (PT) and activated partial thromboplastin time (aPTT). However, these tests have a wide range of sensitivity to each DOAC, depending on the reagent, equipment, and drug [37].

Table 4 presents the anticoagulation effects of different DOACs when measured using different assays [9,12,17,18,25,39,41]. APTT is sensitive to dabigatran, while PT is more sensitive to FXa inhibitors (rivaroxaban, edoxaban) than aPTT. The PT and aPTT are insensitive to apixaban in therapeutic concentrations [25], both being prolonged at only supratherapeutic concentrations [29,37].

Quantitative and specific assays that can assess the effects of dabigatran are diluted thrombin time (dTT), ecarin clotting time, the ecarin chromogenic assay [25], or the chromogenic anti-IIa assay [17,25,39,41]. These tests correlate closely with dabigatran levels measured by the reference standard method, liquid chromatography tandem mass spectrometry [25]. For the quantification of FXa inhibitors, a chromogenic anti-FXa assay calibrated with the specific drug should be used [17,25,41]. When unavailable, an anti-FXa assay calibrated with low-molecular-weight heparin (LMWH) or unfractionated heparin can be useful for excluding clinically relevant levels of FXa inhibitors if they are below the lower limit of activity [25,41] or to determine their presence if higher [34], but this assay cannot be used for drug quantification [25,41]. However, quantitative specific assays for DOAC are not widely available, particularly on an emergency basis [25].

A simple point-of-care (POC) approach using patients’ urine samples, such as a DOAC dipstick test (DOASENSE test), has been shown to be able to accurately identify the presence, absence, and type of DOAC [36,42]. Its best feature is its high negative predictive value, which allows the presence of a DOAC to be rapidly excluded [36,42,43]. In fact, this approach has already been included in some guidelines [12,44] and is a matter of interest in emergency clinical practice.

Standard and modified viscoelastic test (VET) assays can be helpful, too [25,36,45,46], but are still lacking sufficient validation considering sensitivity and specificity compared with DOAC-specific assay [36], and there are no general recommendations currently available [9,25,36]. However, VET assays reveal other concomitant hemostasis disorders which may be the cause or an aggravator of bleeding, allowing for a more accurate hemostasis management [36].

#### 3.2.4. DOAC Cut-Off Level

In some hospitals, cut-off points are used to facilitate rapid decision making [34]. The ISTH guidance [39] and other authors recommend using >50 ng/mL as a threshold for antidote administration for patients with severe/life-threatening bleeding, and >30 ng/mL for patients requiring emergency surgery or invasive procedures [13,25,39]. Below these thresholds, bleeding is not considered to be related to DOACs [39]. Others consider a cut-off drug level of > 30 ng/mL to suggest DOAC reversal during uncontrolled life-threatening bleeding or the need for urgent surgery or invasive procedures [17]. However, some authors maintain that the optimal cut-off drug level is uncertain [11]. The multidisciplinary group involved in this current consensus strategy for DOAC reversal decided to consider a specific DOAC level > 50 ng/mL for life-threatening bleeding or emergency surgery or invasive procedure and a level >30 ng/mL for emergency neurosurgery, neuroaxis anesthesia, or posterior eye chamber emergency surgery or intervention. Usually, in patients arriving at an emergency department, it is difficult to know if they are undergoing a trough or peak in the drug concentration, especially if there is no information available about the timing of their last dose intake. For this reason, we consider that all the DOAC concentrations we are referring to are trough values. However, when an urgent decision is required, waiting for laboratorial results should be avoided. Although DOAC-specific assays may not be available within the time window needed for emergency decisions to be made concerning the management of life-threatening bleeding [34], they should be considered practicable since 25% of the patients in the ANNEXA-4 trial had sub-therapeutic drug levels; however, the real-world reality may be higher [11].

### 3.3. DOAC Reversal

Recommendations for the management of bleeding associated with the use of DOACs vary depending on the agent, the clinical status of the patient, and the capabilities of the institution [24]. Unique patient characteristics such as advanced age, other medications (especially antiplatelet agents), and comorbidities (e.g., renal or hepatic dysfunction), patients’ stability (clinical data such as hemodynamic instability with shock index ≥ 1,0), time since last DOAC intake, or alcohol abuse [14,17,41], must be recognized and considered in the assessment and management of patients with bleeding or requiring emergency invasive procedures [5,14,16,17,41], along with baseline coagulation assays and product availability [16]. The management of bleeding in patients taking DOACs is guided by the site and severity of the bleeding [17]. During severe or life-threatening bleeding, prompt DOAC reversal is a key component of multimodal therapy in addition to supportive measures [16].

Table 5 present the cases in which the Anticoagulation Forum [12] and the SCC of Anticoagulation of the ISTH [39] guidance state that DOAC reversal agents should be reserved.

The actual options available for the reversal and management of DOAC in urgent situations such as bleeding and surgery or invasive procedures include agents both specific (idarucizumab for dabigatran and andexanet alfa for direct FXa inhibitors) and nonspecific (prothrombin complex concentrate [PCC] and activated PCC [aPCC]) agents. Reversal agents should not be used in elective surgery, when bleeding can be managed with local hemostatic measures, when bleeding has stopped, in GIBs that respond to supportive measures, with high drug levels (overdose) without associate bleeding, or when the need for surgery or intervention can be delayed long enough to permit drug clearance [17,25,36,39]. If the bleeding situation is not critical and the clinical context allows for anti-FXa or anti-IIa activity within 60 min, specific tests to guide DOAC emergency management decisions, such as specific reversal, should be used [36]. In contrast, patients presenting with severe bleeding events requiring immediate decision making, bleeding control associated with DOAC reversal should be started as soon as possible after arrival at the emergency department (ED) [36]. In these scenarios without information about previous drug intake or comorbidities, simple urine POC DOAC dipstick tests can provide a rapid answer about the presence or absence of a DOAC [36]. Additionally, a modified VET device (ClotPro^®^) can provide DOAC quantifications [36]. However, none of the DOAC POC tests have been validated in a bleeding scenario.

The decision to reverse anticoagulation should consider the benefit–risk ratio of supporting hemostasis and potentially promoting post-reversal thrombosis [16,18]. The decision to reverse the DOAC is typically based on the severity of the bleed, the expected benefit, and whether clinically relevant plasma drug levels are likely to be present [17]. Urgent reversal of the anticoagulation action of a DOAC is warranted in trauma, emergency surgery, or invasive procedures with a high risk of bleeding (e.g., epidural), and emergency situations (e.g., acute stroke in patients who are candidates for thrombolysis) [17,47].

#### 3.3.1. Reversal Agents

##### Idarucizumab (Praxbind^®^)

Idarucizumab is the specific reversal agent for dabigatran [24,48]. It is a humanized monoclonal antibody fragment with a binding affinity that is approximately 350-fold more potent than dabigatran’s affinity for thrombin [29,48,49]. Idarucizumab reverses the effect of dabigatran in a dose-dependent manner [2]. Its effects start in less than 5 min and its half-life is around 45 min in patients with normal kidney function [17,24], and only 4% of the plasma peak concentration of idarucizumab remains after 4 h [17].

In the RE-VERSE AD (Reversal of Dabigatran Anticoagulant Effect with Idarucizumab), an open label and single-arm study that enrolled 503 patients with uncontrolled bleeding or undergoing urgent invasive procedures, idarucizumab (fixed-dose iv infusion of 2 × 2.5 g aliquots [9,50] over 5–10 min each [9], and within 15 min of each other [2,39]) rapidly, sustainably, and safely reversed the anticoagulant effect of dabigatran [47,49]. Among patients with bleeding, hemostasis was achieved in 68% of the patients within a median time of 3.5–4.5 h, depending on the location of the bleed [12,25,50]. In this study, there was a low number of side effects [2,36] and the rate of thrombotic events was 4.3% 30 days post-reversal of dabigatran activity [25,48,50], with two thirds of these events occurring before resuming antithrombotic therapy [25,50]. The 30-day mortality rate was 13%. In those undergoing procedures or surgery, hemostasis was normal in 92% of patients during their procedure [25,50]. In the REVERSE-AD study, about one quarter of patients experienced a rebound of plasma dabigatran levels 24 h after idarucizumab infusion [12,50]. Indeed, 23–25% of patients may have detectable dabigatran plasma levels or experience a re-elevation in plasma dabigatran levels within 12–24 h (or 18–30 h) after antidote administration [17,24,36,44]. The probability of this rebound effect is higher in patients with very high plasma concentrations at baseline and in patients with kidney disease (creatinine clearance [CrCl] < 50 mL/min) [2,17,36,44]. These patients may require an additional dose of idarucizumab 5 g if there is re-bleeding [17,36].

In 2015, idarucizumab was approved by the United States (US) Food and Drug Administration (FDA) and by European Medication Agency (EMA) for dabigatran-treated adult patients when a reversal of anticoagulation is needed due to life-threatening or uncontrolled bleeding, and when an emergency surgery or urgent invasive procedure is needed, according to the REVERSE AD phase III prospective cohort study [2,10,12,14,16,24,25,37,47,48,50,51,52,53,54,55,56]. In the REVERSE-AD study, the subgroup of trauma patients with bleeding also demonstrated a complete (effective and rapid), and well-tolerated reversal of dabigatran with a single dose of idarucizumab regardless of injury mechanism, age, comorbidity, renal status, hemodynamic stability, or group assignment [47]. Accordingly, idarucizumab has been recommended (class I, level of evidence B) for the reversal of dabigatran in the event of life-threatening or uncontrolled bleeding [56]. If possible, hemodialysis can be considered, reducing plasma dabigatran levels by more than 50% [36]. A systematic review and meta-analysis described a 4% risk of thromboembolism after idarucizumab [8], which was comparable to that in patients receiving 4F-PCC [8,17].

##### Andexanet Alfa (ONDEXXYA™)

Andexanet alfa (coagulation FXa recombinant, inactivated-zhzo) is a modified recombinant inactive form of human FXa acting as a decoy FXa molecule, designed specifically to reversibly bind and sequester FXa inhibitor molecules, rapidly reducing anti-FXa activity through the temporary inhibition of their anticoagulant effects, as they are not able to bind to endogenous FXa [7,17,22,23,40,51,52,57,58]. Furthermore, andexanet alfa also acts as a decoy molecule for the heparin–antithrombin-activated complex, rendering heparin ineffective [7,22]. Andexanet alfa binds with a high affinity, and sequesters and inactivates direct (rivaroxaban, apixaban, edoxaban) and indirectly (heparin, fondaparinux) FXa inhibitors through its interaction with activated antithrombin III [47], restoring thrombin generation in a dose-dependent manner [2,17,23,47,48,51,52]. Andexanet alfa binds to the tissue factor pathway inhibitor (TFPI) to form a non-productive andexanet–TFPI complex, thus inhibiting and reducing TFPI activity [47] and increasing tissue-factor-initiated thrombin generation [2,47]; there is a transient increase in the levels of prothrombin fragments (F1+2), thrombin–antithrombin complexes, and D-dimer [2,39], which normalize within 24–72 h [2].

The ANNEXA-A and ANNEXA-R trials [59] were randomized, double-blind, placebo-controlled studies designed to evaluate the efficacy and safety of andexanet alfa for the reversal of anticoagulation with apixaban or rivaroxaban in older healthy volunteers aged between 50 and 75 years. Anti-Xa activity was rapidly reduced, compared with a placebo, and andexanet alfa rapidly restored FXa activity and thrombin generation, reduced unbound apixaban and rivaroxaban concentrations in treated older participants, and was not associated with serious adverse or thrombotic events.

In the ANNEXA-4 trial, andexanet alfa reversed the anticoagulant activity of the FXa inhibitors, as was demonstrated by a 92% reduction in anti-FXa activity and restoring endogenous thrombin generation with an excellent or good hemostasis 12 h after infusion [58]. Hemostasis was good or excellent in 83% and 80% of apixaban- or rivaroxaban-treated patients, respectively [58]. Andexanet alfa provided a rapid decrease in anti-FXa activity within two minutes [26], had an elimination half-life between 3–4 h [6,60] and 4–7 hours [22,24], and a pharmacodynamics effective half-life of about 30–60 min [6,17,22,60]. For this reason, it is administered as a bolus followed by an infusion [22]. Dosage may vary depending on the time since last intake, the dose of the FXa inhibitor, and the agent used [24,40]. Notably, there was no correlation between nadir FXa activity and bleeding [12,25,58]. At 30-day follow-up, 10% had experienced a thrombotic event, the majority of which occurred before resuming anticoagulation [58].

Andexanet alfa was approved in May 2018 by the US FDA [12,58] and in April 2019 by the EMA [60] after a fast-track approval procedure [7]. Andexanet alfa is specifically indicated for adult patients treated with a direct FXa inhibitor (apixaban or rivaroxaban) when the reversal of anticoagulation is needed due to life-threatening, critical, or uncontrolled bleeding [1,2,5,6,10,12,14,16,25,39,40,44,47,52,54,55,57,60,61], such as ICH or exsanguinating GIB [8]. However, a warning was issued by the US FDA for the potential risk of venous and arterial thromboembolism events, ischemic risk, cardiac arrest, and sudden death [2,12,40,47]. Due to the possibility of these serious adverse effects, these patients must be continually monitored for thromboembolic events [2,47], and anticoagulation must be initiated as soon as is appropriate [25,47].

Although the administration of andexanet alfa in surgical patients is still off-label [7], three case reports described a reduced resistance to the heparin response after its administration in patients undergoing a cardiopulmonary bypass [7,62], and endovascular repair for a ruptured abdominal aortic aneurysm [63]. Because andexanet alfa reverses heparin, if it is administrated before interventions using heparin anticoagulation, it can promote heparin resistance or unresponsiveness, particularly in cardiovascular [17,22] and vascular surgeries [17,20,63]. Therefore, it is recommended not to use andexanet alfa prior to heparin therapy [22].

The results of randomized trials, observational studies, and meta-analyses suggest that andexanet alfa presents a high efficacy in hemostasis control resulting in a reduced mortality, but is associated with an increased risk of thrombotic complications [23]. Local robust protocols for andexanet alfa developed with appropriate centralized decision are important to ensure consistent and proper use whilst avoiding delays, considering it is a new drug with an uncertain risk–benefit trade-off and significant costs [11].

##### Prothrombin Concentrate Complex (PCC)

PCC with four factors (4F-PCC) contains factor II, VII, IX and X, protein C and S, and heparin [5,17,24]. 4F-PCC is indicated for the urgent reversal of an acquired coagulation factor deficiency induced by VKA therapy in adults with acute major bleeding or in those needing urgent surgery or invasive procedures [47]. PCC likely overcomes the anticoagulant effect of DOAC by enhancing thrombin generation through the provision of high concentrations of these coagulation factors [17], namely FII and FX [18].

Although PCC has some ability to reverse abnormal laboratory parameters (PT and endogenous thrombin), it may be associated with a risk of thrombotic complications when used for the reversal of FXa inhibitors [8]. In addition, it has been shown to restore thrombin generation only at low levels of FXa inhibitors [8]. The thrombotic complication rates are reportedly between 4–6.2% [17,24], 0–8% [16], and 2–11% [1]. Treatment with PCC or aPCC increases the concentration of several coagulation factors, including prothrombin which has the longest half-life of about 60 h [44]. Thrombin generation may, therefore, be enhanced for several days after the use of PCC to treat or to prevent major bleeding in trauma or in the perioperative setting [44]. This may increase the risk of arterial and venous thromboembolic complications after treatment with these agents [18,44].

The efficacy and safety of PCC for the different DOACs is difficult to evaluate because no randomized controlled trial has been performed [36]. 4F-PCC was generally effective for achieving hemostasis in patients with major bleeding, and appeared to be associated with a mortality benefit versus no DOAC reversal treatment in patients with traumatic ICH [16]. Others consider their effect on mortality and disability in patients with ICH to be minimal, in addition to their role in limiting the extent of bleeding [18]. Although specific reversal agents are preferred, the role of PCC in DOAC reversal has been evaluated, and multiple studies have demonstrated its potential role as a DOAC reversal strategy [1]. If andexanet or idarucizumab are not available, it is reasonable to use hemostatic agents such as PCC or aPCC (25–50 IU/Kg) [1,10,13,24,25,36,37,38,64].

#### 3.3.2. Clinical Studies About FXa Inhibitors Reversal

Table 6, Table 7 and Table 8 present published clinical studies on FXa inhibitor reversal using different reversal agents. PCC was considered to be an effective and safe alternative for the management of major bleeding in patients on rivaroxaban or apixaban, with around 65–93% of the patients achieving a good hemostatic effectiveness in the first 24 h (Table 6) [65,66,67]. Concerning safety outcomes during the first 30 days after admission, thrombotic events occurred in between 0% and 3.6–8% of the patients and death in 11–32% of the patients using PCC (Table 6) [65,66,67].

An ex vivo study in blood samples from 10 healthy volunteers revealed a hemostatic reversal of rivaroxaban using high-dose 4F-PCC with a similar efficacy to andexanet alfa in flow chamber experiments, but only andexanet alfa restored thrombin generation to baseline levels [68]. Studies comparing andexanet alfa versus PCC for FXa inhibitors reversal revealed significantly lower in-hospital (*p* < 0.01) and 30-day (*p* < 0.001) mortality rates across all bleed types [6,21,61,69], but mostly in ICH bleeding [61] (Table 7). In conclusion, the results of the studies using andexanet versus PCC in FXa inhibitor reversal suggest that differences may exist between reversal and replacement agents for DOAC-related bleeding [58,61,70], and support guideline recommendations about andexanet alfa as the preferred agent for treating FXa inhibitor-related bleedings over 4F-PCC (Table 7) [6,12,16,21,22,25,61,70].

In a meta-analysis of reversal agents for severe DOAC-related bleeding, involving 60 studies with 4735 patients, Gómez-Outes et al. [8] (Supplementary Table S1), showed a high rate of effective hemostasis for FXa inhibitors, being similar either with 4F-PCC (80.1%) or specific reversal agents (80.7%), and a relatively high rate of mortality (17.7%), mostly in ICH (20.2%) [8]. The risk of death after severe DOAC-related bleeding remains significant despite a high rate of effective hemostasis with reversal agents [8]. The rates of thromboembolism were particularly high with andexanet alfa (10.7%) [24]. The rebleeding rate was 13.2%, occurring mainly as ICH (82%), and 78% of rebleeds occurred after the resumption of anticoagulation [8]. Another systematic review and meta-analysis evaluating andexanet alfa (*n* = 438) and PCC (*n* = 1278) for FXa inhibitor-related bleeding, involving a total of 21 studies, revealed similar results with the two agents [68]. Neither reversal agent was significantly associated with an increased effectiveness or a higher rate of venous thromboembolic events [71].

Table 8 presents two posterior multicenter prospective cohort studies from Conolly et al. [58] and Milling et al. [60] where andexanet alfa markedly reduced anti-FXa activity (92–94% [58,60]), and was associated with good or excellent hemostatic efficacy at 12 h in 82% [58] or 80% [60] of patients. There was a significant correlation between hemostatic efficacy and lower mortality in all patients (*p* < 0.001) [60]. A reduction in anti-FXa activity from baseline to nadir significantly predicted hemostatic efficacy in patients with ICH and correlated with a lower mortality in patients under 75 years of age [60]. Thrombotic events during the first 30 days of follow-up occurred in 10% of patients [58,60], but in around 7% of patients who were diagnosed before restarting any anticoagulats [58]. The data suggest the importance of the prompt resumption of anticoagulation after acute major bleeding, when possible and indicated, in these highly prothrombotic patients [60]. No antibodies or neutralizing antibodies to FX and FXa or to andexanet alfa developed [58,60]. In a small retrospective study (*n* = 21) conducted by Nederpelt et al. [34] to evaluate FXa inhibitor-associated extracranial bleeding reversal with andexanet alfa, the authors reported poor overall outcomes.

Considering all data, well-designed prospective randomized controlled trials are needed to further evaluate the effects of reversal therapy on FXa inhibitor-associated bleeding [8,34,71].

**Table 6:** FXa inhibitors reversal using PCC.

Parameter Evaluation	Shulman et al., 2018 [65] Canada (*n* = 66)	Majeed et al., 2017 [66] UPRATE st.; Sweden (*n* = 84)	Last et al., 2024 [67] Germany (*n* = 78)
**Anticoagulant type**	FXa inhibitors: RIV-56%; APIX-44%	FXa inhibitors: RIV-53.6%; APIX-46.4%	DOAC (*n* = 44): APIX: 52%; RIV: 32%; EDOX: 7%; DAB: 9%; VKA (*n* = 34)
**Reversal agent; 1st dose**	PCC: 2.000 IU (fixed dose)	PCC: median 2.000 IU <65 Kg-1.500 IU; >65 Kg-2.000 IU	PCC: DOAC-43%; VKA-79% Idaruzicumab-2/4 patients
**Time last from dose FXa** **Inhibitor to PCC. Median, h**	16.9 (12–21) RIV-18.1; APIX-17.8	12.5 (9–16)	NA
**Exclusion for poor prognosis**	DNR order given	DNR order given	NA
**Age (y), Mean (SD)**	76.9 (10.4)	75 (10.9)	Global: 76.6; DOAC: 75.5 (43–94); VKA: 76.5 (46–91)
**Male sex**	42 (67%)	48 (57%)	Global: 60%; DOAC: 48%; VKA: 74%
**Creatinine clearance on** **admission, mL/min**	<30: 4 (6%) 30–60: 18 (27%)	NA	ARF on admission: Global: 6% DOAC: 5%; VKA: 9%
**Indication for** **anticoagulation**	AF: 54 (82%)	AF: 63 (75%)	AF- Global: 74%; DOAC: 77%; VKA: 71%
**Bleeding** **or/and** **surgeries types**	**Bleeding:** ICH–36 (55%); GIB–16 (24%); Intraspinal–2 (3%); RP–3 (5%); IM-2 (3%); Other–7 (11%); Trauma-related–25 (38%)	**Bleeding:** ICH–59 (70%); GIB–13 (15.5%); Visceral–5 (6%); Musculoskeletal–3 (3.5%); Genitourinary–4 (4.8%); Traumatic–26 (31%)	**Emergency surgery** (within 24 h): DOAC (7%), VKA (21%) with major or clinically relevant non-major bleeding at surgical site until day 30 (*p* = 0.093). **Trauma surgery**: Global: 74%; DOAC: 55%; VKA: 21%
**Criteria for major bleeding**	Critical organ–43 (65%) Overt bleeding: -Transfusion ≥ 2 U–12 (18%) -Hb drop ≥ 2 g/dL–28 (42%)	ISTH criteria [19,34]	Need of RBC transfusions: VKA: 47%; DOAC: 32% (*p* = 0.24) No hemostatic treatment: VKA (3%); DOAC (30%) (*p* = 0.002).
**Hemostatic effectiveness** **(1st day = 24 h)**	Good–43 (65%) Moderate–13 (20%) Poor/None–10 (15%)	Effective- 58 (69.1%) Ineffective- 26 (30.9%) (16/26 with ICH)	Good-Global: 90%; DOAC: 93%; VKA: 85%. DOAC required less prohemostatic treatment than VKA (*p* = 0.002).
**Safety outcome during** **30 days after admission**	TE–5 (8%) Death–9 (14%)	TE–3 (3.6%) Death–27 (32%)	TE–none. Death–Global: 13%; DOAC: 11%; VKA: 15% (*p* > 0.20)

Legend: AF, atrial fibrillation; APIX, apixaban; ARF, acute renal function; DAB, dabigatran; dL, deciliter; DNR, do-not-resuscitate; DOAC, direct oral anticoagulant; EDOX, Edoxaban; g, gram; GIB, gastrointestinal bleeding; ICH, intracranial hemorrhage; IM, intramuscular; ISTH, International Society of Thrombosis and Hemostasis; IU; international units; *n*, number of patients involved; NA, not applicable; PCC, prothrombinic complex concentrate; RIV, rivaroxaban; RP, retroperitoneal; st, study; TE, thromboembolic events; U, unit; VKA, vitamin K antagonists; Y, years.

**Table 7:** FXa inhibitors reversal using andexanet alfa versus PCC.

	Sutton et al., 2023 [21] USA (*n* = 255)	Dobesh et al., 2023 [70] USA (*n* = 4395)	Coleman et al., 2021 [6] USA (*n* = 3.030)	Cohen et al., 2022 [61] UK (*n* = 410)
**Study type**	Multicenter, retrospective, observational		Matched 2 database [57,66]	
**Reversal agent**	**ANDEX–PCC** (*n* = 85)–(*n* = 170)	**ANDEX–4F-PCC** (*n* = 2122)–(*n* = 2273) Low dose–2.200 IU (68,8%) (median)	**ANDEX–PCC** (*n* = 342)–(*n* = 733)	**ANNEX–4** [57]–**ORANGE** [66] **ANDEX only–PCC only** (*n* = 322)–(*n* = 88)
**Anticoagulant type**	APIX: 78.8–47.7% RIV: 18.8–14.7% EDOX: <5–0 ENOX: <5–37.7%	APIX: 59. 9–62.3% RIV: 40.1–37.7%	APIX: 47–51% RIV: 50–41% EDOX: 3–8%	APIX: 55–NA RIV: 36–NA
**Age, y, mean**	76.1–71.8	65.6–66.6	69.1–70.1	77.7–74.9
**Male sex**	100–97.7%	57.2–60.5%	55–50%	53–49.5%
**Atrial Fibrillation**	87.1–72.9%	NA	NA	83.9–78.9%
**Bleed Type:** **-GIB** **-ICH** **-Other** **-Trauma**	45.9–52.9% 29.4–28.8% 24.7–18.2% Not described	56.8–59.9% 31.4–29.1% 1.8–1.8% 50% of all ICH	40–41% 20–23% 9–32% 31–4%	25.5–28.6% 64.9–67.1% 9.6–4.4% Not described
**In-hospital Mortality**	10.6–25.3% (*p* = 0.01) 30-day: 20–32.4% (*p* = 0.039)	6.0–10.6% (*p* < 0.01) ICH: 12.6–23.3% GIB: 2.5–4.3%	4–10% ICH: 9–25% GIB: 1–4% Trauma: 4–7%	30-day: 14.6–4.1% (*p* < 0.001) ICH: 15.3–48.9% (*p* < 0.01) GIB: 12.2–25.0% (*p* = 0.10) Others–16.1–12.5%
**Door-to-needle time, h**	NA	Mean: 8.2–7.3 Median: 2.5–2.3	NA	NA
**Time since last anticoagulant dose**	NA	<8 h: 44.1–41.5% 8–18 h: 41.8–41.1% >18 h: 14–17.4%	NA	NA
**Study** **conclusion**	Patients treated with andexanet alfa for FXa inhibitor-related major bleeds had significantly lower in-hospital and 30-day mortality rates compared to 4F-PCC	The odds of in-hospital mortality were 50% lower with andexanet alfa vs. 4F-PCC (*p* < 0.01) and the risk reduction was similar for ICH (45%) and GIB (51%) (both with *p* < 0.01).	In-hospital mortality differed by bleed type (highest in ICH-22.7%; lowest in GIB-3.9%) and agent administrated. Andexanet alfa was associated with the lowest rate of in-hospital mortality across all bleed types.	Adjusted 30-day mortality rates were lower for those treated with andexanet alfa than in matched patients receiving PCC (*p* < 0.001). In the ICH, those treated with Andexanet alfa had lower mortality than patients receiving PCC. (*p* < 0.01)

Legend: ANDEX, andexanet alfa; APIX, apixaban; EDOX, edoxaban; ENOX, enoxaparin; F, factor; GIB, gastrointestinal bleeding; h, hour; ICH, intracranial hemorrhage; IU, international unit; *n*, number of patients involved; PCC, prothrombinic complex concentrate; RIV, rivaroxaban; UK, United Kingdom; USA, United States of America; Y, year.

**Table 8:** FXa inhibitors reversal using andexanet alfa.

	Conolly et al., 2019 [58] (*n* = 352) (ANNEXXA-4)	Milling et al., 2023 [60] (*n* = 479)	Nederpelt et al., 2020 [34] (*n* = 21)
**Study type**	Multicenter, prospective, open-label, single-group trial. (USA, Eur)	Multicenter, prospective, open-label, single-group trial. (USA, Eur, Can, Japan)	Retrospective, consecutive case series at two hospitals. (USA)
**Reversal–ANDEX.**	Mainly low dose (82.7%)	Dose NA	Mainly low dose (85.7%)
**Anticoagulant type**	RIV–36%—EDOX–3% APIX–55%—ENOX–6%	RIV–37%—EDOX–8% APIX–51%—ENOX–5%	RIV–33.3% APIX–66.7%
**Age, y, mean (±SD)**	77.4 ± 10.8	78.0 ± 10.9	73.2 ± 15.4
**Male sex**	53%	54%	61.9%
**AF; VTE**	AF–80%; VTE–17%	AF–81.2%; VTE–15%	AF–76.2%; VTE–4.8%
**Kidney dysfunction** (% of patients)	GFR < 30 mL/min–9%	GFR < 30 mL/min–9.2%	GFR < 30 mL/min–0% AKI–33.3%; CKD–42.9%
**Median time since last dose #**	NA	11.4 h	<18 h: 84.2%; >18 h: 15.8%
**Bleed Type**	ICH–64%; GIB–26%; Other-10%	ICH–69%; GIB–23%; Other-8%	ICH–0%; GIB–23.8%; Other-42.8%; Trauma-23.8%
**Median decrease in anti-FXa activity** from baseline to nadir after ANDEX	APIX: ↓ 92% → ↓ 32% at 12 h RIV: ↓ 92% → ↓ 62% at 12 h ENOX: ↓ 75%	APIX: ↓ 93%; ENOX: ↓ 75% RIV: ↓ 94%; EDOX: ↓ 71% ↓ all 2 min. after ANDEX	NA
**Hemostasis efficacy**	Excellent or good at 12 h after ANDEX in 80% of patients. GIB–85%; ICH–80%	Excellent or good in 80% of pts. GIB–82%; ICH–79%; other-82%; RIV-81%; APIX-79%; EDOX-79%; ENOX-88%.	Excellent or good: 47,6% of pts: Excellent–14.3% Good–33.3%; Poor–52.4%
**30-day mortality**	14% (*n* = 39)	15.7% (*n* = 75) ICH–16.9%; GIB–11.9% ≥75 y: 19.6%; <75 y: 6.8%	38.1% (*n* = 8) Bleeding related to trauma and surgery
**30-day TE**	10% (*n* = 34)	10.4% (*n* = 50)	19% (*n* = 6)
**Resume** anticoagulation *	62%	67.4%	NA
**Biomarker-** **efficacy correlation**	Reduction in anti-FXa activity was not predictive of hemostatic efficacy. However, it was modestly predictive in pts with ICH (AUC of 0.64)	No significant association between anti-FXa activity change and hemostatic efficacy, and mortality. Significant correlation between hemostatic efficacy and lower mortality in all pts (*p* < 0.001).	NA
**Study conclusion**	Andexanet alfa markedly reduced anti-FXa activity and 82% of patients had excellent or good hemostatic efficacy at 12 h after ANDEX.	ANDEX: ↓ anti-FXa activity was associated with good or excellent hemostatic efficacy in 80% of pts. ↓ of anti-FXa activity significantly predicted hemostatic efficacy in ICH and correlated with lower mortality in pts under 75 years old (*p* = 0.02)	Poor overall outcomes, a low rate of hemostatic efficacy, and a high rate of TE and mortality for extracranial bleeds compared to previously published data.

Legend: #, last anticoagulant dose; * until 30 days; ↓, decrease; AF, atrial fibrillation; ANDEX, andexanet alfa; AKI, acute kidney injury; APIX, apixaban; Can, Canada; CKD, chronic kidney disease; EDOX, edoxaban; ENOX, enoxaparin; Eur. Europe, GFR, glomerular filtration rate; h, hour; *n*, number of patients involved; NA, not available; pts, patients; RIV, rivaroxaban; TE, thrombotic events; USA, United States of America; y, years; ≥, equal or greater than; >, greater than; <, less than.

#### 3.3.3. Guidelines for FXa Inhibitors Reversal

Most guidelines (Supplementary Table S2) advocate for the use of specific antidotes, to reverse the effects of DOACs, particularly in life-threatening settings [16,22,72]. All guidelines advocate for the use of idarucizumab as a first-line therapy to reverse dabigatran in life-threatening bleeding and/or if an emergency surgery/invasive procedure is needed [5,9,10,12,14,25,39,41,44,49,53,54,55,56,64,73,74,75,76,77,78,79,80,81,82,83].

Andexanet alfa is indicated for the reversal of FXa inhibitors as a first-line therapy in life-threatening bleeding, especially for apixaban and rivaroxaban by several national and international guidelines across multiples scientific disciplines [5,9,11,12,14,22,23,25,26,41,44,45,49,54,56,64,72,75,76,77,78,79,80,82,83,84,85]. In addition to DOAC holding, first-line therapy with either andexanet alfa or 4F-PCC in major/life-threatening bleeding has been suggested by some guidelines for apixaban, rivaroxaban [10,44,55,74,81], and also for edoxaban [10,12] and betrixaban [12], although the use of the last two FXa inhibitors is off-label. The 2019 Anticoagulation forum [12] suggests andexanet alfa for major apixaban- and rivaroxaban-associated bleeding or patients who have undergone prior emergency surgery, although its use is off-label in the last case. At least six guidelines recommend 4F-PCC as a first-line therapy for edoxaban reversal [16,44,53,54,73,83].

Despite being off-label, most recent guidelines suggest the use of nonspecific agents, including 4F-PCC or aPCC (25–50 IU/Kg), when specific reversal agents are not available, in cases of serious life-threatening bleeding [16,22,72]. Before anticoagulation reversal is considered, it is essential to assess patient indication for anticoagulation and the underlying thrombotic risk to anticipate the timing and dose of thromboprophylaxis as well as full anticoagulation resumption following anticoagulation reversal [22]. The high cost and the risk of complications (thrombotic events) require special care when using andexanet alfa, and some experts recommended documenting its administration in a dedicated national registry [23].

## 4. DOAC Reversal Algorithm in Urgent Clinical Scenarios

**Figure 1:**
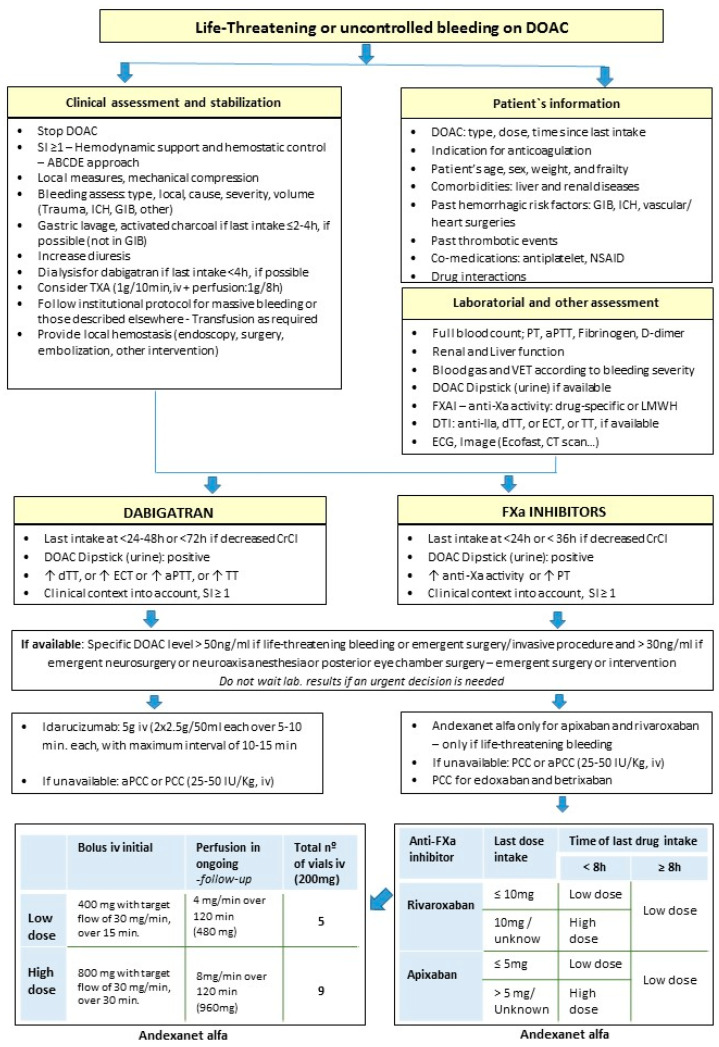
DOAC reversal algorithm in life-threatening or critical bleeding. Legend: ABCDE, Airway–Breathing–Circulation–Disability–Exposure approach; aPTT, activated partial thromboplastin time; CrCl, creatinine clearance; CT, computed tomography; DOAC, direct oral anticoagulant; DTI, direct thrombin inhibitor; dTT, diluted thrombin time; ECG, electrocardiogram; ECT, ecarin clotting time; FXAI, Factor X inhibitors; g, gram; GIB, gastrointestinal bleeding; h, hour; ICH, intracranial hemorrhage; IU, international unit; iv, intravenous; Kg, Kilogram; lab., laboratorial; LMWH, low-molecular-weight heparin; mg, milligram; mL, milliliter; min., minutes; ng, nanogram; NSAID, nonsteroidal anti-inflammatory drugs; PCC, prothrombinic complex concentrate; PT, prothrombin time; SI, shock index; SICH, spontaneous ICH; TT, thrombin time; TXA, tranexamic acid; VET, viscoelastic tests.

**Figure 2:**
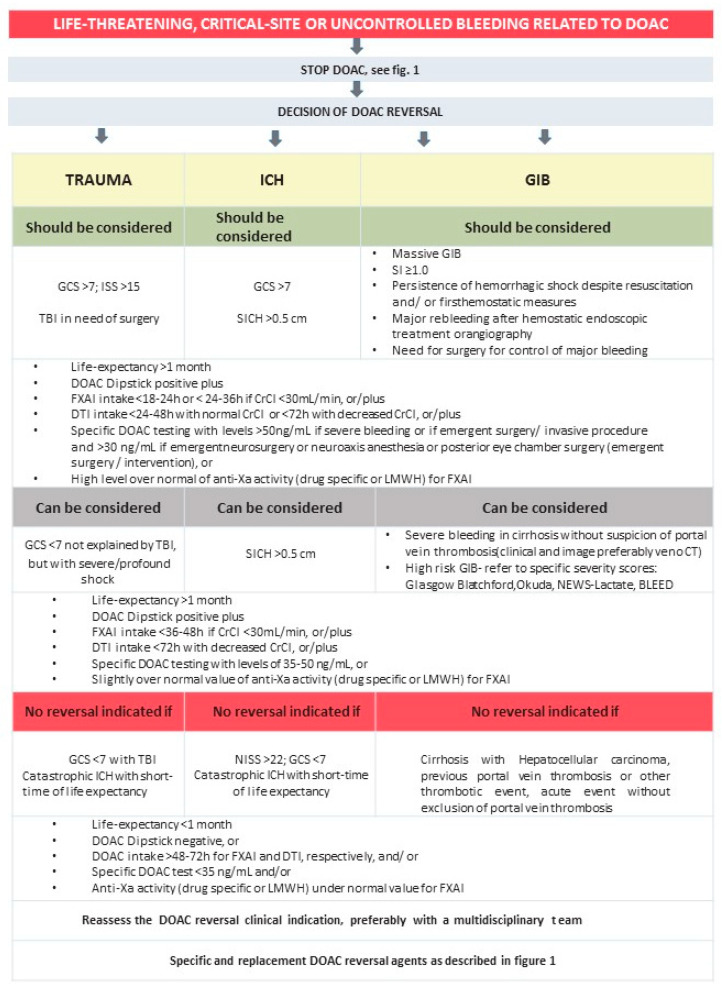
DOAC reversal in life-threatening bleeding in predefined clinical settings. Legend: ≥, equal or greater than; anti-Xa, anti-factor X activated; cm, centimeter; CrCl, creatinine clearance; CT, computed tomography; DOAC, direct oral anticoagulant; DTI, direct thrombin inhibitor; Fig., Figure; FXAI, factor X inhibitors; GIB, gastrointestinal bleeding; GCS, Glasgow Coma Score; h, hour; ICH, intracranial hemorrhage; ISS, injury severity score; LMWH, low-molecular-weight heparin; ml, milliliter; min, minutes; ng, nanogram; NISS, new ISS; SI, shock index; SICH, spontaneous ICH; TBI, traumatic brain injury.

A proposal for the reversal of DOAC activity is described in Figure 1 and Figure 2. The termination of any anticoagulation therapy should be performed immediately in every unknown critical bleeding patient [36]. Immediate supportive care (resuscitation, stabilization, local hemostatic measures) is critical for all patients whether or not a replacement or reversal agent is used [5,25], as described in Figure 1 and elsewhere [25,29,44,45,53,54,86]. Physicians should not only focus on the effects of anticoagulation on bleeding severity and outcome [36]. The use of VET according to institutional protocols may be useful [29,54,86], particularly in patients with liver disease, because in this setting PT and aPTT may not be reliable measures of hemostatic function [25]. Hypothermia, acidosis, hypocalcaemia, a depletion of fibrinogen and other coagulation factors, and reduced platelets, as well as hyperfibrinolysis, need immediate treatment [36].

There are some critical considerations for managing the bleeding associated with anticoagulation: if the bleeding event is life-threatening and if the site is critical, the agent, dose, and time since last intake [5,14,44], if comorbidities exist (e.g., hepatic/renal failure), the concurrent use of potential hemorrhagic drugs (aspirin or P2Y12 inhibitor; non-steroidal anti-inflammatory drugs (NSAID), or parenteral agents (e.g., enoxaparin for VTE in oncologic patients [5]), previous thromboembolic events, patient’s age and frailty, and drug interactions [5]. Patients should be monitored for 2–5 half-lives of the applicable agent according to renal and liver function, drug pharmacokinetics, comorbidities, and clinical status [5,36]. The drug half-lives based on CrCl are as follows: the half-life of dabigatran in patients with CrCl between 50 and 79 mL/min, 30 and 49 mL/min and 15 and 29 mL/min is 15, 18 and 27 h, respectively [25]. Concerning FXa inhibitors, the half-lives of apixaban, edoxaban, and rivaroxaban with CrCl ≥ 30 mL/min are 6–15 h while that of of betrixaban is 19–27 h [25]. With s CrCl of 15–29 mL/min, the half-life of apixaban and edoxaban is 17 h [25], and it is 11–15 h for rivaroxaban. In patients with severe renal dysfunction, laboratory evaluation to detect residual anticoagulant activity (e.g., dabigatran is 80–85% renally excreted) is recommended [12,25].

In major and life-threatening bleeds, or if emergency surgery is needed, all anticoagulants must be discontinued and reversal agents must be administrated, if available, along with the usual care in these settings [44,48]. Clinical and laboratorial assessments of patients must be carried out at admission as described in Figure 1 and Figure 2, and further individualized hemostatic protocols have already been described elsewhere [29,54,86]. Critically ill, high-risk patients have dynamic clinical courses and require frequent reassessments, especially after treatment with a replacement or reversal agent [5], and if they are not responding as expected (reassess: laboratory, VET, or imaging studies) [5]. In all cases, laboratory monitoring is recommended, although in urgent situations, the results should not be waited for [44].

The specific reversal agents are idarucizumab for the rapid reversal of dabigatran in life-threatening bleeding or emergency surgery/invasive procedures [10,24,25,45,50] and andexanet alfa for FXa inhibitors (rivaroxaban, apixaban) in life-threatening, critical, or uncontrolled bleeding [10,24,25,45,48]. If specific reversal agents are not available, 4F-PCC (at dose of 25–50 IU/Kg) [1,10,13,24,25,29,36,37,38,45,48] or aPCC can be considered for both types of DOAC [25,29]. PCC should only be preferred if the severity of blood loss suggests a depletion of coagulation factors (possibly confirmed by VET), for which factor supplementation by PCC is superior [36]. In contrast, if the DOAC plasma concentration is low, a watch-and-wait strategy is preferable [36].

To make a decision about intravenous thrombolysis in stroke patients with a history of any DOAC intake, the current recommendations vary between 30 and 50 ng/mL or an anti-Xa activity of less than 0.5 IU/mL as cut-off for the safety of thrombolysis [36].

While reversal is important in the context of major/life-threatening bleedings, the risk of subsequent thromboembolic events due to reversal, ranging from 7.2 to 12% within 30 days from the event, should also be kept in mind [48]. The incidence of these events is lower with idarucizumab (4%) [8].

### 4.1. Bleeding Management in Patient Under DOAC in Special Clinical Situations

#### 4.1.1. Trauma

Injured patients taking an oral anticoagulant account for 4% of all trauma patients presenting to emergency departments (EDs) [85]. Given the increase in DOAC usage, as well as the increase in trauma in geriatric patients, who are more likely to be on anticoagulation medication for a comorbidity, we can expect an increase in trauma injuries (including traumatic brain injury [TBI]) in patients on DOACs [35,85,87], and a higher mortality than in those who are not taking an anticoagulant [85]. Several studies have confirmed equivalent or improved outcomes with DOAC use in non-head-injury trauma and a lower overall mortality in trauma with ICH when compared with VKA [35,85].

In DOAC-treated patients who present with trauma without bleeding, the Anticoagulation Forum suggest against the routine use of reversal agents, as is also the case for patients undergoing a DOAC overdose without bleeding [12]. If the patient is found to have traumatic bleeding or requires an invasive procedure, the administration of a reversal agent may be warranted depending on the severity of the bleed or the urgency of and risk of bleeding during the procedure [12].

Once clinically relevant bleeding has been detected, an assessment of hemodynamic stability must be carried out [85], as well as an evaluation of the type, cause, location, and severity of bleeding [85], comorbidities (kidney and liver diseases), patient’s age and frailty, and drug history (anticoagulant, antiplatelet, and others promoting bleeding). In the case of a history of DOAC intake, we must consider the type and dose of anticoagulant (half-life, mode of elimination), the time since last intake, and the existence of chronic disorders (kidney or liver diseases) [45,87].

Trauma guidelines [54] recommend that the degree of hypovolemic shock and transfusion requirements should be assessed using the shock index (SI) and/or pulse pressure (grade 1C) [54]. Management includes early identification, anticoagulation reversal, and damage control [41]. As life-saving measures stopping the anticoagulant effect, hemodynamic support with fluid resuscitation and blood products, mechanical compression, tourniquet application, surgical, and radiological intervention (intravascular embolization), or endoscopy should be considered to identify and treat the cause of bleeding [45,85,87], followed by a reversal of the anticoagulants (specific reversal or, if not available, nonspecific replacement) [85,87] (Figure 1 and Figure 2). Beyond the ABCDE approach and supportive measures [87], we can consider, if possible, decreasing the plasma concentration of dabigatran through hemodialysis [38] (about 50–60% can be removed during a 4 h procedure) and the concentration of FXa inhibitors through hemoperfusion with Cytosorb filters [45] (Figure 1). If DOAC ingestion was within the past 2–4 h, oral activated charcoal can be administered [38], if clinically possible (not on GIB).

Once coagulopathy is identified, individualized treatment should be considered in a retrograde way, following the advanced trauma life support concept of “treat first what kills first” [45]. Accordingly, we should first stabilize the clot by blocking hyperfibrinolysis; second, we should improve the clot firmness, and third, we should improve thrombin generation [45]. Antifibrinolytic therapy including tranexamic acid should be the first-line treatment, taking an empiric approach within 3 h after injury [45]. Fibrinogen, platelets, and FXIII are the main determinants of clot firmness [45,86]. Fibrinogen is the first coagulation factor to decline to a critical level below 2 g/L during a massive hemorrhage [45,86]. Therefore, fibrinogen supplementation should be performed early on to improve clot firmness and to reduce transfusion requirements [45,86]. Further individualized hemostatic protocols have already been described elsewhere [45,54,86].

Laboratory screening (coagulation, blood count, liver and renal function, and blood gasses) may help to identify the clinical situation, and estimate the potential accumulation and the remaining duration of drug effects [45].

In the presence of ongoing bleeding, with normal tests (PT, aPTT, fibrinogen, VET testing and eventually platelet function) and the exclusion of mechanical reasons for bleeding, a residual effect of DOAC should be considered by measuring the activity of calibrated anti-Xa (for rivaroxaban, apixaban, edoxaban), anti-IIa, or dTT (for dabigatran) [35,45] to guide the administration of reversal agents [35]. The 2023 European trauma guidelines suggest the measurement of dabigatran plasma levels using dTT, and if this is not possible or available, using standard TT in patients treated or suspected of being treated with dabigatran (grade 2C) [54]. They also suggest the measurement of plasma levels of anti-FXa inhibitors in patients treated or suspected of being treated with one of these agents (grade 2C) using anti-Xa activity calibrated for the specific agent, and if unavailable, they suggest LMWH-calibrated anti-Xa assays as a reliable alternative (grade 2C) [54].

It should be taken into account that female sex, increased age, decreased height and weight, and lower estimates CrCl can be associated with higher anti-Xa levels at admission [35].

Almost all patients with TBI require an immediate reversal of anticoagulation in addition to initial resuscitation (hemodynamic management, airway stabilization as needed), a neurological examination, and a head computed tomography (CT) on admission [85]. The incidence of delayed ICH is about 0.8% [85]. Patients who are on anticoagulants and sustain an ICH have a higher risk of death and hematoma expansion, with rapid anticoagulant reversal usually being necessary for even small, relatively asymptomatic hemorrhages to prevent hematoma expansion [85]. Prompt and aggressive anticoagulation reversal is important for long-term outcomes in patients with anticoagulant-related ICH [85].

The 2023 European trauma guidelines stated that, if bleeding is life-threatening in those receiving dabigatran, idarucizumab (5 g iv) is recommended (grade 1C) [54]. Reversal with andexanet alfa is suggested (grade 2C) by these guidelines if the bleeding is life-threatening in the presence of an apixaban or rivaroxaban effect, especially in patients with TBI [54]. They also suggest the administration of PCC (25–50 U/kg) (grade 2C) if andexanet alfa is not available, or in patients receiving edoxaban [54].

It should be noted that the concomitant use of PCC and andexanet alfa might increase the risk of thromboembolic complications due to the increased thrombin generation potential [88]. The need for DOAC reversal must be weighed against thrombotic risks [87,88].

#### 4.1.2. Spontaneous Intracerebral Hemorrhage

A spontaneous intracerebral hemorrhage (SICH) is the most common form of ICH and is responsible for up to 27.9% of all strokes. However, despite being less frequent than ischemic stroke, its toll in terms of deaths and loss of disability-adjusted life years is equivalent [89]. This apparent paradox is, at least in part, due to the lack of effective treatments: whereas in ischemic stroke reperfusion therapy has revolutionized patient care [90,91], no intervention has shown such efficacy in ICH patients. In fact, treatment for SICH patients is, in many aspects, largely supported by and extrapolated from ischemic stroke studies [49]. Nevertheless, there are interventions that have been shown to have an impact on SICH outcomes. In the acute setting, these interventions focus on hematoma expansion and formalized care bundles.

Hematoma expansion occurs in 20–40% of SICH patients and is associated with a worse prognosis. It is estimated that for every 1 mL increase in hematoma volume, the chances of dying or becoming dependent increase by 7% [92]. Two interventions have been recommended to address and prevent hematoma expansion, anticoagulation reversal and blood pressure (BP) control [49]. Since hematoma expansion is an early event [93], these interventions should be delivered as soon as possible to patients, and should be seen as the SICH equivalent of thrombolysis and thrombectomy for ischemic stroke: the sooner they are delivered the better, with more of the brain being saved [94]. Several observational studies have confirmed this paradigm within SICH care, with patients being treated earlier with BP control and anticoagulation reversal showing a better prognosis and lower rates of hematoma expansion [95,96]. Regarding BP control, the guidelines recommend lowering BP for patients presenting with a systolic blood pressure (SBP) above 150 mmHg, with a target BP of 140 mmHg [49]. Lower values are not recommended, as no benefits were found in terms of clinical outcomes and a higher rate of adverse events was observed in the ATTACH-2 trial [97]. No specific recommendations have been made regarding the antihypertensive drugs to be used. Commonly used drugs in clinical practice include nicardipine, urapidil, and labetalol. Nitroprussiate is also highly effective, but should probably be used with caution, and only in patients with severe or refractory hypertension due to its vasodilatory effects which might aggravate intracranial pressure [98]. Regarding oral anticoagulation reversal, all patients with SICH on anticoagulant therapy should immediately stop the drug and be assessed for anticoagulation reversal eligibility. The choice of reversal agent depends on the anticoagulant associated with the ICH. 4F-PCC is an effective reversal agent for coumarin-associated SICH and should be preferred instead of plasma, as it restitutes hemostasis faster [99]. PCC is also an effective nonspecific reversal agent for DOAC-associated bleeds [100] but new specific agents have been developed, namely idarucizumab [100] and andexanet [101,102]. Recently, the ANNEXA-I randomized controlled trial directly compared andexanet alfa with the standard-of-care treatment (mostly PCC) in ICH patients who were taking factor Xa inhibitors. In this study, the administration of andexanet resulted in a higher hemostatic efficacy and in a better control of hematoma expansion than usual care but was associated with thrombotic events, including ischemic stroke [103].

An algorithm to guide oral anticoagulation reversal is provided in Figure 1 and Figure 2. The initial approach for these patients typically consists of a brief clinical history and a physical examination. Clinical history should focus on time of symptom onset, vascular risk factors, and the medication the patient is taking, particularly antithrombotics. Information regarding the timing of the last intake of these drugs should be promptly gathered, as this might affect the decision to use reversal agents. Given that ICH is a life-threatening disease, a physical exam should follow the typical ABCDE approach, with an emphasis on the Glasgow Coma Score (GCS) and National Institutes of Health Stroke Scale (NIHSS) on (D)isability evaluation. Typical laboratory evaluation includes a complete blood cell count, renal and liver function tests, and coagulation tests, along with CT or magnetic resonance imaging (MRI) to confirm the diagnosis of cerebral hemorrhage. Upon confirmation of diagnosis, BP should be lowered to below 140 mmHg, if necessary, and consideration should be given to anticoagulation reversal. If necessary, specific tests can be performed to assess anticoagulation status, but such tests should not delay reversal agent administration for patients at risk of hematoma expansion presenting early after their last drug intake.

Once the patient has been stabilized and a diagnosis has been confirmed, ICH-specific care bundles should be rapidly initiated [104]. Beyond rapid anticoagulant reversal and intensive BP reduction, the major components of a care bundle for ICH include neurosurgical intervention and the implementation of well-defined criteria for surgical evacuation and/or the insertion of an external ventricular drain, as well as temperature and glucose control. ICH patients should be admitted to a dedicated stroke unit as soon as possible, since ICH is a dynamic event with a high risk of early (48 h) clinical deterioration [105]. Depending on the clinical condition and the specific organization of different hospitals, ICH patients may also be admitted to an intensive care unit or a neurocritic care unit.

**Figure 3:**
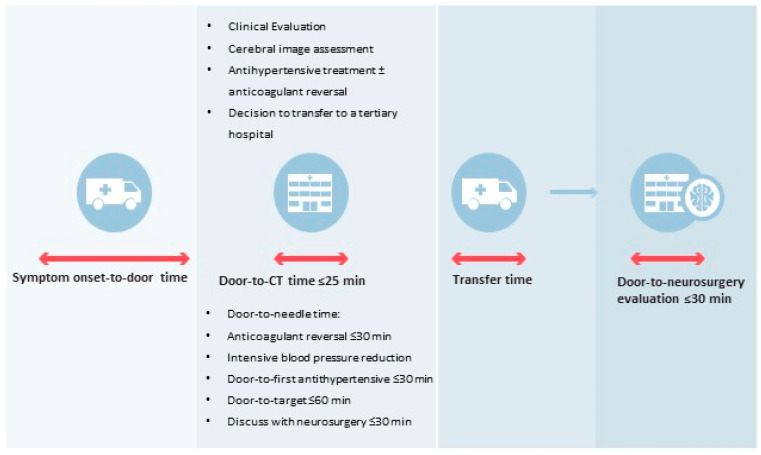
Timeline of a well-organized regional telestroke network for spontaneous intracerebral hemorrhage. Legend: fig, figure; lab., laboratorial; min., minutes; SBP, systolic blood pressure.

Finally, acute care bundles should incorporate the definitions of specific time metrics. Given the lack of consensus regarding recommended process targets, the values presented here should be considered to be merely indicative (Figure 3).

Door-to-CT scan: ≤25 min;

Door-to-needle time (anticoagulant reversal): ≤30 min

Door-to-needle time (intensive blood pressure reduction): Door-to-first antyhipertensive ≤ 30 min; door-to-target ≤60 min.

Looking ahead, if the use of mobile stroke units (MSUs) becomes more widespread, it is plausible that the initial interventional approach could be shifted to the prehospital setting. This is supported by the recent INTERACT 4 trial [105], which found that very early BP control in the ambulance (within 2 h of symptom onset) was associated with a decreased odds of poor functional outcome among patients with ICH. The feasibility of initiating anticoagulant reversal therapy in the MSU setting is currently unknown.

#### 4.1.3. Gastrointestinal Bleeding

GIB is the most common major bleed in DOAC-treated patients and accounts for more than 50% of all DOAC-related major bleeds; fortunately, it has a lower mortality than ICH. The rate of major GIB with DOAC is 3.3% [106].

The management of GIB has multiple and parallel targets: triage, risk stratification, general supportive measures such as fluid resuscitation and blood transfusions, the management of coagulopathies, anticoagulants and antiplatelet agents (cessation, reversal and resuming), adequate timing for endoscopy, and the management of rebleeding. It’s treatment needs a skillful and timely approach from multiple specialties using multiple resources for the correct diagnosis and bleeding control, such as imunohemotherapy, a gastrointestinal endoscopy, radiology, radionuclide imaging, and surgery.

Clinicians can use various scores for the risk assessment of GIB, and some have previously been described for upper GIB such as the Rockall score (age, shock, comorbidities), the Glasgow–Blatchford score (GBS-blood urea nitrogen, Hb, SBP, and others such as heart rate, liver disease and cardiac failure), or AIMS65 (albumin, international normalized ratio (INR), altered mental status, SBP, age older than 65 years) [107]; others have been developed for lower GIB such as the Oakland score, Strate, NOBLADS (NSAID use, no diarrhea, no abdominal tenderness, BP ≤ 100 mm Hg, antiplatelet non-aspirin drug use, albumin < 3.0 g/dL, disease score ≥ 2 according to the Charlson Comorbidity Index, and syncope) [108,109], and others have been developed for any type of GIB such as the National Early Warning Score + Lactate (NEWS-L) or BLEED (ongoing bleeding, low SBP, elevated PT, erratic mental status, and unstable comorbid disease) [110,111].

European and American societies subscribe to the concept of THE timely reversal of anticoagulation and the timely use of an endoscopy for bleeding source control. Concerning warfarin, the American Society of Gastrointestinal Endoscopy [112] recommends that an endoscopy should not be delayed in patients with serious GIB and an INR < 2.5 (low-quality evidence recommendation). Also, the American College of Gastroenterology (ACG), state that, in patients with GIB with an INR > 2.5, anticoagulant reversal agents should be considered prior to endoscopy [113]. Although the evidence is very low quality, prior to or concomitant with the administration of reversal agents, endoscopic hemostasis may occur without delay in patients with an INR of 1.5–2.5 [113]. The European Society for Gastroenterology (ESGE) guidelines on endoscopy diagnosis and the management of GIB recommends DOAC reversal in massive acute upper GIB with hemodynamic instability or life-threatening bleeding (severe ongoing bleed), using a specific reversal agent or PCC. In the case of hemodynamic instability or life-threatening bleeding, an early endoscopy and the reversal of anticoagulation must be performed [114]. The position of various societies on DOAC reversal is consistent about its use in severe and life-threatening situations but is less clear about the methods that should be employed for the reversal. The ACG-Canadian Association of Gastroenterology, in their Clinical Practice Guidelines in 2022, stated that PCC should not be used for the management of DOAC during acute GIB and in the peri-endoscopic period; for patients under dabigatran, they suggest against the use idarucizumab, and for patients under rivaroxaban or apixaban, they suggest against andexanet alfa administration [84]. More recently, other societies express different approaches to the problem of DOAC reversal. The Spanish Society of Digestives Diseases and the Spanish Society of Thrombosis and Haemostasis suggest, for massive and life threatening nonvariceal GIB and DOAC, the use of idarucizumab for dabigatran and, if unavailable, the use of PCC and for rivaroxaban; for apixaban (and edoxaban off -label), they suggest the use of andexanet afa and PCC if andexanet afa is unavailable [41]. An update to the ACG guideline on acute lower GIB indicates DOAC reversal in severe cases with hemodynamic instability despite initial resuscitation, a life-threatening bleed that does not respond to initial resuscitation, and the cessation of the anticoagulant alone. Specific reversal agents are suggested when available if the DOAC has been taken within the past 24 h. The most relevant subgroup analyses are the RE-VERSE AD trial and ANNEXA-4 trials [80]. Targeting can be carried out by testing DTI for the use of idarucizumab, or anti-FXa levels for the use of andexanet alfa for apixaban/rivaroxaban if the last intake of the drug was within < 24 h. At present, there is no definite role for PCC in the reversal of FXa inhibitors. The data indicate higher mortality of patients treated with PCC. On the basis of data from the ANNEXA-4 study [58], andexanet alfa is recommended by the National Institute for Healthcare and Clinical Excellence (NICE) in patients with life-threatening bleeding including acute GIB who are treated with either apixaban or rivaroxaban.

### 4.2. Recommendation About Monitoring the Efficacy and Safety on DOAC Reversal

Reappearance of anticoagulant activity of anti-FXa drugs may occur after stopping the infusion of andexanet alfa (rebound of anti-Xa within 2 h of infusion completion [1]) and, less frequently, within 12–30 h after reversal of dabigatran with idarucizumab [1,2,17,24,36,44,52].

Monitoring of andexanet alfa should be based mainly on clinical parameters indicative of an appropriate response (e.g., achievement of hemostasis), lack of efficacy (e.g., re-bleeding) and adverse events (e.g., thromboembolic events) [22].

Whilst DTI and FXa inhibitors can prolong aPTT and PT, respectively, normal results cannot be used to established lack of anticoagulant activity [48]. If adequacy of anticoagulation needs to be assessed, dTT and anti-FXa levels, respectively, are recommended [48].

However, monitoring of andexanet alfa should not be based on anti-FXa activity [22]. Commercially available anti-FXa activity assays are not suitable for measuring anti-FXa activity after andexanet alfa administration, given the falsely high levels of anti-FXa activity that occur, which results in a significant underestimation of the andexanet alfa reversal activity [22]. Indeed, the ANNEXA-4 trial demonstrated that a reduction in anti-FXa activity in blood correlates poorly with hemostatic effectiveness in extracranial bleeding [58].

### 4.3. Timing of Anticoagulation Resumption After Major Bleeding

After reversal, anticoagulation must be resumed as soon as clinically indicated, if the patient‘s clinical condition is safe and if proper hemostasis is achieved [22,25].

The decision to resume anticoagulant therapy after a bleeding event is critically important and should balance the risk of re-bleeding in the case of resumption, and the risk of thromboembolism if anticoagulation is not resumed [5,13,25,48]. This decision needs to be made case-by-case, by a multidisciplinary team, through a thorough assessment of the risks and benefits [9,13]. The timing of restarting anticoagulation involves balancing the severity, location, and consequence of the bleeding event with the indication of anticoagulation, associated thrombotic risk, and possibility of re-bleeding, if the patient’s clinical condition is stable, and if hemostasis was achieved [5,22,25,55]. If and when resuming oral anticoagulation is needed, a multi-parametric choice should be kept in mind [52].

After a bleeding event, it is crucial to assess the bleeding risk using bleeding scores. These scores include non-modifiable, potentially modifiable, and modifiable risk factors [14,115,116,117,118]. Whenever possible, modifiable and potentially modifiable risk factors should be corrected prior to resuming antithrombotic therapy, namely hypertension, impaired renal function, alcohol abuse, anemia, thrombocytopenia, and significant drug interactions that potentially increase DOAC levels. It is very important to choose the most suitable and correct anticoagulant dosing according to patients’ characteristics (age, weight, renal and hepatic function, drug interactions) and indication for anticoagulation.

The decision to restart anticoagulation is well defined in several guidelines [25]. In conditions with a high thrombotic risk, the recommendation is for the early resumption of anticoagulation once hemostasis is achieved and the patient is clinically stable; for patients with a moderate or high re-bleeding risk, individualized strategies are more appropriate [25]. Keep in mind that the 30-day mortality rate from AF without oral anticoagulation is about 25% after an ischemic AF stroke [25].

Anticoagulation needs to be resumed in a timely manner, considering that bleeding risk outweighs thrombotic risk when anticoagulation is resumed early after the bleeding event [9,13]. Furthermore, thrombotic events after anticoagulant reversal may be due to the patient’s intrinsic prothrombotic state, the bleeding scenario, or the reversal agent [5]. However, the specific timing of when to resume anticoagulation is not well defined [13]. The 2021 European Heart Rhythm Association guidelines suggested a net assessment in favor of resuming anticoagulation and to resume DOAC as early as clinically feasible [9].

#### 4.3.1. Trauma

The 2023 European trauma guidelines [54] recommend the early initiation of mechanical pneumatic compression (IPC) which should be intermittent while the patient is immobile and has a bleeding risk (grade 1C). They also recommend combined pharmacological and IPC thromboprophylaxis within 24 h after bleeding has been controlled and until the patient is mobile (grade 1B) [54].

#### 4.3.2. Spontaneous Intracerebral Hemorrhage

In patients taking OAC who experience an SICH, two key questions arise: should anticoagulation be resumed, and if so, when. A potential approach is to categorize patients based on their bleeding risk. OAC is generally considered to be a relative contraindication following SICH when the underlying cause cannot be rectified and the risk of recurrence is elevated.

Patients with AF and a moderate-to-high bleeding risk, as determined by the HAS-BLED score [119], as well as those with probable Cerebral Amyloid Angiopathy according to the Boston criteria version 2.0 [120], fall into this category. In such cases, endovascular Left Atrial Appendage Closure might be a suitable alternative, given its feasibility, safety, and non-inferiority to DOAC [121]. For the remaining patients, determining the optimal timing to restart OAC following an ICH is a complex issue without a definitive answer. Most guidelines recommend a waiting period post-ICH, but individual assessment is crucial. Patients with a high risk of thrombotic events, such as those with a mechanical heart valve, may benefit from early OAC resumption. Conversely, in patients with non-valvular AF where the risk of thromboembolic events outweighs the risk of recurrent ICH, restarting OAC approximately 7–8 weeks post-ICH might be considered [49].

#### 4.3.3. Gastrointestinal Bleeding

The characteristics of the bleeding event influence the risks associated with restarting antithrombotic therapy, including bleeding location, whether the bleeding cause was identified and treated, and whether further surgical or procedural interventions are planned [25,122].

Bleeding risk is dynamic, and evaluation of bleeding risk factors should be made at every patient contact, towards improved efficacy and safety of antithrombotic therapy [123].

The timing of resuming anticoagulation and the type and dose of anticoagulant should be evaluated. A prospective study which evaluated the benefits and risks of resuming anticoagulation after GIB recommended resuming within 2 weeks after the GIB event to achieve the best net clinical benefit [124].

### 4.4. Importance of the Multidisciplinary Team

A multimodal approach appears to be the optimal strategy to restore hemostasis in patients with bleeding and/or coagulopathy attributed to DOAC [24]. The management of acute major bleeding in patients under DOAC is well established [25], but there is a lack of standardized protocols about how and when to resume anticoagulant therapy after hemostasis is achieved [13]. International guidelines recommend the development of a hospital-based multidisciplinary approach including cardiology, gastroenterology, neurology, intensive care, hemostasis, neurosurgery, and vascular surgery specialists [9] to provide an individualized optimal balance between benefit and risk [13]. The outcome of patients with oral anticoagulant-related bleeding can be improved not only by the use of reversal agents, but also by multidisciplinary and multimodal support applied in a timely and integrated strategy [12,13,52]. Ideally all hospitals should have a multidisciplinary “bleeding team” [52] and a “thrombosis team” [5,9,13].

## 5. Discussion

This multidisciplinary expert consensus developed a stepwise guidance framework for DOAC reversal in the event of life-threatening bleeding and emergency surgery/procedures, including a narrative review of the most recent scientific literature. The immediate reversal of these agents if life-threatening bleeding occurs is indicated in an emergency setting [10], and is still the primary concern with DOAC use due to the significant morbidity and mortality associated with bleeding if it is not treated immediately and effectively [2].

Every hospital should develop a protocol for bleeding management in patients taking anticoagulants, including DOAC, and an indication for their reversal [39,52].

DOAC-associated bleeding involves an initial assessment, a decision to replete or replace and repair, and finally to resume anticoagulation [85]. A time-to-treat analysis to determine the therapeutic window during reversal agents could be useful [85]. Most of the recommendations are based on expert opinion and interpretation of available evidence [18].

The reversal antidotes exhibited a significant restoration of normal hemostasis in more than 90% of the patients with major DOAC-associated bleeding, with a well-tolerable safety profile and a low mortality rate [125]. Real-world experience with specific antidotes is still limited [125]. The off-label use of PCC has been the mainstay of DOAC reversal, in addition to support measures [2]. Despite the recommendations, the lack of a comparator arm in the REVERSE-AD and ANNEXA-4 trials, the reports of thrombotic events in treated study subjects, and the high cost of andexanet alfa relative to PCC generate concerns [5,50,57,58,125].

The Anticoagulation Forum stated “with the recent advent of potentially life-saving, but also costly and potentially prothrombotic DOAC reversal agents, it is imperative that clinicians and institutions be prepared to use these agents in a manner that is both cost-effective and optimizes patient outcomes” [12].

There are real and potential challenges associated with DOAC reversal strategies that may be broadly grouped into acquisition and cost, operational logistics (storage, preparation), and appropriate and timely administration [12,39]. Given the high cost and prothrombotic potential of DOAC reversal agents, judicious use of these agents is essential [12,39]. Centralized, controlled access is prudent to optimize the appropriate treatment (e.g., indication, dose, administration) of patients and the storage of samples (e.g., refrigeration, light protection) [12]. Most importantly, the best way to treat a bleed related to DOAC is to prevent it by using the right drug at the right dose in the right patient, and by withdrawing anticoagulation in patients without an indication for such therapy [51].

No solid evidence about DOAC reversal is available, and many current guidelines’ recommendations/suggestions are mainly based on panelists’ judgments [52]. Further studies assessing the use of specific antidotes in real-world practice are needed [125]. Post-marketing surveillance and registries are needed to better determine their clinical utility, particularly in special circumstances such as reversal before thrombolytic therapy in patients with acute ischemic stroke, or additional dosing if there is incomplete reversal with ongoing bleeding [39].

Finally, the resumption of anticoagulation must take place as soon as possible after the benefits outweigh the patient’s bleeding and thrombotic risks.

## 6. Conclusions

We developed a multimodal and multidisciplinary DOAC reversal management algorithm supported by the most recent literature, expert’s opinion, and guidelines from several scientific societies within different areas of medicine for the assessment and treatment of bleeding patients. All hospitals should have a multidisciplinary team to provide guidance on bleeding and thrombosis management, and to better support patients with oral anticoagulation. The real-world experience with the reversal of specific DOACs is still limited. Their judicious use is essential because of the high cost and prothrombotic risk. Further prospective trials assessing the use of specific antidotes are needed, as well as a national registry for retrospective evaluation in the real world.

## Figures and Tables

**Table 1:** Definitions of massive bleeding/transfusion events [27,29,30,31].

Concept	Definition
**Loss of blood**	One TBV in 24 h or > 50% of one TBV in 3 h
**Ongoing bleeding**	150 mL/min or 1.5 mL/Kg/min in 20 min
**Number of transfused units of RBC**	GIB—3 units of RBC in 1 h ≥10 units of RBC in 24 h 4 units of RBC in less than 4 h plus hemodynamic instability, and anticipated ongoing bleeding.

Legend: GIB, gastrointestinal bleeding; h, hour; min, minutes; RBC, red blood cells; TBV, total blood volume; ≥, Equal or greater than; >, greater than.

**Table 2:** ISTH and SCC of Anticoagulation Criteria for the definition of major bleeding in non-surgical patients [19,32].

Definition Components
(a) Fatal bleeding;
(b) And/or symptomatic bleeding in a critical area or organ, such as intracranial, intra-spinal, intraocular; retroperitoneal, intra-articular or pericardial, or intramuscular with compartment syndrome;
(c) And/or bleeding causing a fall in Hb levels of ≥2 g/dl, or leading to the transfusion of ≥2 units of RBC

Legend: dl, deciliter; g, gram; Hb, hemoglobin; ISTH, International Society of Thrombosis and Hemostasis; RBC, red blood cells; ≥, equal or greater than.

**Table 3:** ISTH prognostic classification of major bleedings according to risk of death within 30 days [33].

Categories of Major Bleeding	Grade	Definition
**Serious**	**I**	Articular or ocular
**Severe**	**II a**	ICH with GCS ≥ 14
	**II b**	Non-ICH major bleeding without shock or hypotension
**Life-Threatening**	**III a**	ICH with GCS < 14 or non-ICH major bleeding with shock or hypotension
	**III b**	Pericardial bleeding

Legend: GCS, Glasgow Coma Scale; ICH, intracranial hemorrhage; ISTH, International Society of Thrombosis and Hemostasis; ≥, equal or greater than; <, less than.

**Table 4:** Effects of DOAC on coagulation screening tests [9,17,25,39,41].

	Normal aPTT (Control: 29)	Prolonged aPTT	Normal PT (Control: 11.6)	Prolonged PT	Normal TT (NV: 14–21 s)	Prolonged TT
**Dabigatran**	May not exclude on-therapy levels, especially if a non- sensitive aPTT reagent is used [9,17,25,39,41]	Suggests presence on-therapy or above on-therapy levels [25,41]			Excludes clinically relevant levels * [25,39,41]	Does not discriminate between clinically significant * and insignificant levels [25]
**Apixaban ^%%^**	Does not exclude clinically relevant levels * [17,25] ^#^		Does not exclude clinically relevant levels * [17,25] ^#^	Suggests supratherapeutic levels [17,25]		
**Rivaroxaban and Edoxaban**	Does not exclude clinically relevant levels * [17,25]		Does not exclude clinically relevant levels * [17,25]	Suggests on-therapy levels at peak or above on-therapy levels [25]		

Legend: aPTT, activated partial thromboplastin time; NV, normal values; PT, prothrombin time; TT, thrombin time; sec, seconds; *, clinically relevant DOAC concentration are levels which may contribute to bleeding or surgical risk [25]; ^#^, particularly in case of apixaban [9,17,39], and if insensitive reagents are used [25] ^%%^, the PT and aPTT are insensitive to apixaban [25].

**Table 5:** Situations in which DOAC reversal agents should be used [12,39].

Life-threatening bleeding or uncontrolled bleeding. Bleed into a critical organ/site or closed space Persistent major bleeding, despite local hemostatic measures, that is not controlled with maximal supportive measures, and there is demonstration or reasonable expectation that the patient has clinically relevant plasma DOAC levels, or risk of recurrent bleeding because of delayed DOAC clearance or DOAC overdose Need for an emergency surgery or intervention associated with a high risk of bleeding that cannot be delayed long enough to allow for drug clearance.

Legend: DOAC, direct oral anticoagulants.

## Supplementary Materials

The following supporting information can be downloaded at: https://www.mdpi.com/article/10.3390/jcm13226842/s1, Table S1: FXa inhibitors reversal using andexanet alfa versus PCC; Table S2: Guidelines for DOAC reversal.
