# Supplementary material for: Urgent Reversal of Direct Oral Anticoagulants in Critical and Life-Threatening Bleeding: A Multidisciplinary Expert Consensus
Source: J Clin Med. 2024 Nov 14;13(22):6842. doi: 10.3390/jcm13226842 (PMC11595216; doi:10.3390/jcm13226842)
Supplement: Supplementary file 1 [file jcm-13-06842-s001.zip › jcm-3293894-supplementary.pdf]

Journal of
Clinical Medicine

MDPI

SUPPLEMENTARY TABLES

Supplementary TableS1. FXa inhibitors reversal using andexanet alfa versus PCC

	**Gómez-Outes *et al*/2021** [8]**, Spain (n=4.735)**
**Study type**	Meta-analysis of 60 studies: Retrospective cohort (n=48); Prospective (n=10);Clinical trials (n=2)
**Reversal agent**	**ANDEX** (n=936)**:** 10 studies; 77,3% with low dose ; **Idarucizumab** (n=1.111): 8 studies; 5g iv;<br>**PCC** (n=2.688): 34 studies; dose of 25-50 IU/Kg
**Anticoagulant type**	APIX-32%; RIB-36%; EDOX- 1% ; Dabigatran- 31%
**Age, y, mean**	77 ±3,5 (range: 68-86)
**Male sex**	57%
**Atrial Fibrillation; VTE**	AF: 82% ; VTE: 14%
**Bleed Type:**	GIB - 26%; ICH - 55%; Others - 19%
**In-hospital Mortality**	-Global: 17,7%% (significant heterogeneity between trials).<br>-ICH: 20,2% (type of bleed also contributed to heterogeneity in mortality rates, which were higher in ICH)<br>-Extracranial: 15,4%<br>-No relevant differences in death rates depending on the reversal agent used, the type of study, risk of bias.
**Time since last antico-agulant dose, h**	n=9 studies<br>Mean: 13,8 ±3,6 (range: 8-21)
**Anticoagulation re-sumption**	After controlling the bleeding event in 57% (range in studies: 25-73%)<br>Time to resume: 11 days (5-27days)
**Thrombotic event rate**	Global-4,6%;<br>ANDEX-10,7%; 4F-PCC-4,3%; Idarucizumab-3,8%.
**Effective hemostasis rate**	Global-78,5%;<br>ANDEX-80,7%; 4F-PCC-80,1%; Idarucizumab-76,7%.
**Rebleeding rate**	13,1% (78% of rebleeds occurred after resumption of anticoagulation; occurred 82% as ICH)<br>Mean time to rebleeding: 13,4 ±9,3 days (range: 5-34)

	**Gómez-Outes *et al*/2021** [8]**, Spain (n=4.735)**
**Study conclusion**	-High rate of effective hemostasis (80%), with 4F-PCC or specific reversal agents, and a relatively high rate of deaths (17,7%). The risk of death after severe DOAC-related bleeding remains significant despite a high rate of effective hemostasis with reversal agents.<br>-Failure to achieve hemostatic efficacy was strongly correlated with a fatal outcome.<br>-Thromboembolism rates are particularly high with andexanet alfa (10,7%)

Legend: AF, Atrial fibrillation; ANDEX, Andexanet alfa; APIX, Apixaban; EDOX, Edoxaban; F, Factor; GIB, Gastrointestinal bleeding; h, hour; ICH, Intracranial hemorrhage; PCC, Prothrombinic complex concentrate; RIB, Rivaroxaban; VTE, Venous thrombotic event; Y, Year

Supplementary Table S2. Guidelines for DOAC reversal

Guideline / year	Clinical context	Guideline or expert consensus recommendations
**Grottke *et al*/ 2024** [44]<br>ESA-OAC Guidelines	Severe bleeding in urgent surgical and nonsurgical settings, including ICH	**DTI:** 1st line: consider Idarucizumab (grade 2C);<br>If unavailable, consider PCC or aPCC (grade 2C-3).<br>**FXAI**: APIX/RIVA: 1st line – suggest andexanet alfa or PCC (grade 2C);<br>If unavailable, consider aPCC (grade 2C); EDOX: consider PCC (grade 3).
**Kietaibl *et al*/ 2023** [53]<br>ESA Guidelines	Severe perioperative bleeding	**DTI:** 1st line: consider Idarucizumab (grade 2C).<br>**FXAI**: APIX/RIVA/EDOX: suggest PCC (25IU/Kg) at first rather than andexanet alfa (grade 2C).
**Rossaint *et al*/ 2023** [54]<br>Trauma Guidelines<br>(2023 European Task Force for ABC in Trauma)	Life-threatening bleeding following trauma	**DTI:** 1st line: recommend Idarucizumab (grade 1C);<br>Measure: dTT, TT (grade 2C).<br>**FXAI**: APIX/RIVA: especially if ICH, suggest andexanet alfa (grade 2C);<br>If unavailable, suggest PCC (25-50IU/Kg) (grade 2C). EDOX: suggest PCC (25-50IU/Kg) (grade 2C). Measure: anti-Xa calibrated for specific agent;<br>if unavailable: anti-Xa (LMWH) activity (grade 2C).
**Baugh *et al*/ 2020** [5]<br>ACEP- expert panel rec. on AC reversal strategies in the ED	Life-threatening or critical bleeding or nonbleeding requiring emergency procedure	Last dose: Dabigatran <18h; APIX/RIVA <18h; EDOX<10-14h; BETRIX<19-24h.<br>**DTI:** 1st line – suggest Idarucizumab for bleeding and previous surgery.<br>**FXAI**: 1st line – suggest andexanet alfa for APIX/RIVA.<br>**DTI/ all FXAI** – If unavailable: 4F-PCC

Guideline / year	Clinical context	Guideline or expert consensus recommendations
**Joglar *et al*/ 2024** [10]<br>ACC/ AHA Joint committee on clinical practices Guidelines	Life-threatening bleeding	**DTI:** 1st line: recommend Idarucizumab (grade 1);<br>If unavailable, suggest aPCC (50IU/Kg once) (grade 2a).<br>**FXAI**: recommend either andexanet alfa (APIX, RIVA, and EDOX) or<br>4F-PCC (25-50 IU/Kg) (grade 1).<br>PCC may be not indicated for patients with TE in the previous 3 months
**Steffel *et al*/ 2021** [9]<br>EHRA Guidelines practical guide	life-threatening, or critical bleeding	**DTI:** consider Idarucizumab (5g iv). Suggest Idarucizumab in moderate to high hemorrhagic risk emergent procedures.<br>**FXAI**: suggest andexanet alfa. Suggest the off-label use of andexanet alfa in life-threatening situations requiring an immediate intervention.<br>**DTI/ all FXAI** – If unavailable: consider PCC (50 IU/Kg; +25 IU/Kg if indicated) or aPCC (50 IU/Kg; maximum 200 IU/Kg/day).
**Van Gelder et al/ 2024** [14] ESC-EACTS	Severe or life-threatening bleeding or urgent surgery	**DTI:** consider Idarucizumab (Class IIA, level B).<br>**FXAI**: suggest andexanet alfa (Class IIA, level B);<br>If unavailable, consider 4F-PCC for APIX/RIVA/ EDOX
**Tibi et al/ 2021** [78]<br>STS/SCA/AmSECT/SABM update to the clinical practice Guideline on PBM	Emergent cardiac surgery (preoperative anticoagulants)	With recent DOAC intake or laboratorial evidence of a DOAC effect, administration of a specific reversal antidote is recommended:<br>**DTI:** recommend Idarucizumab (Class IIA, level C-LD).<br>**FXAI**: recommend andexanet alfa for APIX/RIVA (Class IIA, level C-LD).<br>**DTI / all FXAI** – If unavailable: recommend PCC (Class IIA, level C-LD), recognizing that the effective response may be variable

Guideline / year	Clinical context	Guideline or expert consensus recommendations
**Cuker** ***et al*****/ 2019** [12]<br>Anticoagulation Forum (ACF) Guidance on Reversal of DOAC	life-threatening, critical-site/organ, or uncontrolled bleeding with maximal supportive measures	and there is demonstration or reasonable expectation of clinically relevant plasma DOAC levels:<br>**DTI:** suggest Idarucizumab (5 g iv). If unavailable, suggest aPCC (50 IU/Kg)<br>**FXAI**: suggest andexanet alfa for APIX/RIVA. If unavailable, suggest 4F-PCC at fixed dose of 2.000 IU; EDOX/BETRIX: suggest off-label use with either high dose andexanet alfa or 4F-PCC (2.000 IU).
	Emergent surgery/invasive procedure with high bleeding risk, that cannot be delayed	and there is demonstration or reasonable expectation of clinically relevant plasma DOAC levels:<br>**DTI:** suggest Idarucizumab (5 g iv). If unavailable, suggest aPCC (50 IU/Kg)<br>**FXAI**: suggest andexanet alfa at the same dosing used for major bleeding;<br>If unavailable, suggest 4F-PCC at fixed dose of 2.000 IU
**Mead** ***et al*****/ 2023** [72]<br>WSO (World Stroke Organization)	Summary: systematic reviews - ICH	**DTI**: recommend Idarucizumab for dabigatran.<br>**FXAI**: recommend andexanet alfa; if unavailable, suggest 4F-PCC.
**Greenberg** ***et al*****/ 2022** [49]<br>AHA/ASA	Spontaneous intracranial hemorrhage	**DTI:** suggest Idarucizumab (5 g iv) (grade 2A, level B-NR);<br>if unavailable, suggest aPCC or 4F-PCC and/or hemodialysis (grade 2b; level C-LD)<br>**FXAI**: suggest andexanet alfa (grade 2A, level B-NR);<br>if unavailable, suggest 4F-PCC or aPCC (grade 2b, level B-NR).

Guideline / year	Clinical context	Guideline or expert consensus recommendations
**Christensen** ***et al*/ 2019** [73] ESO Guideline	Acute Intracranial hemorrhage	**DTI**: 1st line - strongly recommend Idarucizumab.<br>**FXAI**: 1st line – recommend andexanet alfa for APIX/RIVA;<br>If unavailable: 2nd line- weak recommendation of 4F-PCC for FXAI (37.5-50 IU/Kg)<br>EDOX: recommend PCC as 1st line (50 IU/Kg).<br>TXA: contraindicated in spontaneous ICH-DOAC reversal.
**Kakkos** ***et al*/ 2021** [82] 2021 ESUS clinical practice guidelines on the management of VTE	Life-threatening or uncontrolled bleeding	**DTI:** 1st line. suggest Idarucizumab (5 g iv)<br>**FXAI**: 1st line: suggest andexanet alfa for APIX/RIVA/EDOX;<br>**FXAI**: If unavailable, recommend PCC (50 IU/Kg, with additional 25 IU/Kg if clinically needed) as 2nd line.
**Sengupta** ***et al*/ 2023** [80] ACG guideline for management of acute lower GIB	Severe lower GIB with hemodynamic instability despite initial resuscitation	life-threatening bleed that does not respond to initial resuscitation and cessation of the anticoagulant alone, reversal is suggested:<br>**DTI:** suggest Idarucizumab, if last intake within the past < 24h.<br>**FXAI**: suggest andexanet alfa for APIX/RIVA if last intake within <24h.<br>At present, there is no definite role for PCC in reversal of FXAI.
**Gralnek** ***et al*/ 2021** [81] 2021 ESGE Guideline on endoscopy diagnosis and management of GIB	Massive acute upper GIB with hemodynamic instability or life-threatening bleeding (severe ongoing bleed)	In case of hemodynamic instability or life-threatening bleeding, must do early endoscopy and reversal of anticoagulation:<br>• Anticoagulant stopped<br>• DOAC reversal with specific agent or PCC should be considered.

Guideline / year	Clinical context	Guideline or expert consensus recommendations
**Milling *et al*/ 2020** [16]<br><br>Review of guidelines	Severe or life-threatening bleeding or prior emergent surgery	• Most guidelines advocate using specific reversal agents as treatment, where available, and particularly if life-threatening bleed.<br>• Despite being off-label, most recent guidelines recommend the use of PCC or aPCC (25-50IU/Kg), when specific reversal agents are not available, in cases of serious life-threatening bleeding<br>• Most guidelines recommend PCC for EDOX reversal<br>• ACF [9] suggest andexanet alfa for APIX/RIVA-associated major bleeding or prior emergent surgery, although andexanet alfa is off-label in the last setting.<br>• ACF [9] also suggest off-label treatment with either high dose of andexanet alfa or 4F-PCC for EDOX/BETRIX-related major bleeding or prior emergent surgery.

Legend: ABC, Atrial fibrillation better care; AC, Anticoagulants; ACC, American College of Cardiology; ACEP, American College of Emergency Physicians; ACF, Anticoagulation Forum; ACG, American College of Gastroenterology; AHA, American Heart Association; AmSECT, American Society of Extracorporeal Technology; Anti-Xa, anti-factor X activated activity; APIX, Apixaban; aPCC, activated PCC; ASA, American Stroke Association; BETRIX, Betrixaban; DOAC, Direct oral anticoagulants; DTI, Direct Thrombin inhibitor- Dabigatran; dTT, diluted thrombin time; EACTS, European Heart Rhythm Association; ED, Emergency department; EDOX, Edoxaban; EHRA, European Heart Rhythm Association; ESA, European Society of Anesthesiology; ESC, European Society of Cardiology; ESGE, European Society of gastrointestinal Endoscopy; ESO, European Stroke Organization; ESUS, European Society for Vascular Surgery; FXAI, Factor X activator inhibitors; g., gram; GIB, Gastrointestinal bleeding; h, hour; ICH, Intracranial hemorrhage; iv, intravenous; IU; International units; Kg, Kilogram; LMWH, Low molecular weight heparin; OAC, Oral anticoagulants; PBM, Patient blood management; PCC, Prothrombin complex concentrate; 4-F-PCC, Four factor PCC; rec., recommendation; RIVA, Rivaroxaban; SABM, Society for the Advancement of Blood Management; STS, Society of Thoracic Surgeons; SCA, Society of Cardiovascular Anesthesiologists; TT, Thrombin time; TXA, Tranexamic acid; TE, Thromboembolic events; US, United States; VTE, Venous thromboembolic event; WSO, World Stroke Organization
